# Separate and Joint Associations of Occupational and Leisure-Time Sitting with Cardio-Metabolic Risk Factors in Working Adults: A Cross-Sectional Study

**DOI:** 10.1371/journal.pone.0070213

**Published:** 2013-08-06

**Authors:** Madina Saidj, Torben Jørgensen, Rikke K. Jacobsen, Allan Linneberg, Mette Aadahl

**Affiliations:** 1 Research Centre for Prevention and Health, Glostrup University Hospital, Glostrup, Denmark; 2 University of Copenhagen, Copenhagen, Denmark; 3 University of Aalborg, Aalborg, Denmark; University of Perugia, Italy

## Abstract

**Background:**

The workplace is a main setting for prolonged sitting for some occupational groups. Convincing evidence has recently accumulated on the detrimental cardio-metabolic health effects of leisure-time sitting. Yet, much less is known about occupational sitting, and the potential health risk attached compared to leisure-time sitting.

**Objective:**

To explore the separate and joint associations of occupational and leisure-time sitting with cardio-metabolic risk factors in working adults.

**Methods:**

All working adults (N = 2544) from the Health2006, a Danish population-based study, were included in this cross-sectional study. Participants reported hours of sitting during work, during leisure-time along with socio-demographic and behavioral characteristics, including physical activity. Cardio-metabolic risk factors (waist circumference, body mass index, body fat percentage, total cholesterol, HDL cholesterol, LDL cholesterol, triglycerides, insulin, hemoglobin A1c and plasma glucose) were measured. Associations were explored by linear regression for leisure-time, occupational, and overall sitting time.

**Results:**

Statistically significant (p<.05) detrimental associations of leisure-time sitting were observed with all cardio-metabolic risk factors, except hemoglobin A1c and plasma glucose. Similarly, occupational sitting time was significantly detrimentally associated with HDL cholesterol, triglycerides, and insulin. For categories of sitting time, a joint adverse association of sitting much during both work-time and leisure-time was observed.

**Conclusion:**

The associations of occupational sitting time with cardio-metabolic risk factors were fewer and weaker compared to leisure-time sitting. Yet, the joint associations of occupational and leisure-time sitting with cardio-metabolic risk factors were higher than the separate. Our findings amplify the need for further focus in this area prior to making assumptions about equivalent health risks across sedentary behaviors. To our knowledge, this is the first study to contrast the deleterious associations of prolonged occupational and leisure-time sitting, both separately and jointly.

## Introduction

Sedentary behaviors, often operationalized as sitting time, are those behaviors that are characterized by low energy expenditure of less than 1.5 METS, either in a sitting or reclining position [1]. In the past decade, hundreds of published studies have measured sedentary behavior as a distinct concept from physical activity, and there is now recognition that the two exert in(ter)dependent influences on health [2]. Sitting is a distinct risk factor for major health outcomes, counting premature all-cause and cardiovascular mortality, cardio-metabolic morbidity, and increased risk of type II diabetes [3]–[5], and some types of cancer [6]. Importantly, the evidence is currently limited by a predominant focus on leisure-time sitting, particularly on TV viewing during leisure-time. However, TV viewing is a specific sedentary behavior that may not be a marker of overall sitting time [2]. Fewer studies have examined time spend in other, yet as common, domains of sitting, such as transport-related sitting time [7], [8], total sitting time [9]–[12], and - of specific relevance for the present study - occupational sitting time [8], [13]–[15]. Because time spent sitting in the workplace is a main contributor to daily sitting time for many working adults, the workplace has lately been identified as a key setting in which to reduce adults’ sitting time to improve health conditions [16]–[18]. Recommendations that workplaces implement strategies to reduce the amount of sitting time have been formulated, some with an expected reduction in chronic diseases, such as diabetes and cardiovascular disease, as a result [17]. However, the majority of evidence pertaining to the health impact of occupational sitting to date comes from the musculoskeletal literature. Evidence on the impact on other health conditions are truly limited [19]. A natural question raised is, whether the health risks are equivalent across domains of occupational and leisure-time sitting. The aim of this study was to explore the separate and joint associations of occupational and leisure-time sitting with cardio-metabolic risk factors in a sample of working adults.

## Materials and Methods

### Ethics Statement

The study was approved by the Ethical Committee of Copenhagen County (KA-20060011). All participants gave their written informed consent prior to their inclusion in the study.

### Design and Participants

Participants comprised all working adults from the Health2006, a Danish cross-sectional population-based study conducted June 2006-June 2008 at the Research Centre for Prevention and Health. The participants in Health2006 were drawn as a random sample from the background population aged 18 to 69 years living in the South-Western part of the greater Copenhagen area. A total of 3471 persons entered the study (participation rate: 44.7%). All participants completed questionnaires on health, lifestyle and social factors, and underwent a health examination with anthropometric measurements and assessment of cardio-metabolic biomarkers. Details of the enrolment and examination procedures are described elsewhere [20].

### Sitting Time

Sitting time was assessed using the Physical Activity Scale 2 (PAS2) [21], a revised version of the Physical Activity Scale (PAS) [22] that was validated against diaries, accelerometry and V02max [22], [23]. In PAS2 participants report hours and minutes spend in usual weekly physical activity and daily sedentary pursuits during leisure-time and at work. Contruct validity was assessed and the questionnaire was tested by cognitive interviewing in adult Danes [21]. The exposures considered in this study were daily *leisure-time sitting* (h/day) derived from the question ‘In your leisure time, how many hours and minutes per day do you spend watching TV, sitting quietly, reading, and listening to music or the like’, *occupational sitting time* (h/day), derived from the question ‘During work, how many hours and minutes per day do you engage in sedentary work’ and *overall sitting time* (h/day) as the sum of the two; all as continuous variables. Furthermore a *categorical* measure of overall sitting within categories of high/low daily occupational and leisure-time sitting, based on cut points at ≤3 and >3 h/day for leisure-time sitting, and <6 and ≥6 h/day for occupational sitting, was included. Categories were 1 = Low Leisure-time (≤3 h/day)/Low Occupational (<6 h/day) sitting, 2 = High Leisure-time (>3 h/day)/Low Occupational (<6 h/day) sitting, 3 = Low Leisure-time (≤3 h/day)/High Occupational (≥6 h/day) sitting, 4 = High Leisure-time (>3 h/day)/High Occupational (≥6 h/day) sitting.

### Cardio-metabolic Risk Factors

Assessed cardio-metabolic risk factors were waist circumference (cm), body mass index, BMI (kg/m^2^), body fat (%), total cholesterol (mmol/l), HDL cholesterol (mmol/l), LDL cholesterol (mmol/l), triglycerides (mmol/l), insulin (pmol/l), hemoglobin A1c, HbA1c (%), and plasma glucose (mmol/l). Health examinations were conducted from 7.00 am to 12.30 pm. Participants were asked to be fasting from midnight prior to the examination.

Waist circumference was measured at midway level between the lowest rib and the iliac crest. Height was measured without shoes to the nearest centimeter, weight was measured in light clothing without shoes to the nearest 0.1 kg and BMI was calculated as kg/m^2^. Body fat was measured by bioelectrical impedance (Tanita model TBF-300). Fasting blood samples were drawn for lipids (determined enzymatically for total cholesterol, HDL cholesterol and triglycerides; LDL cholesterol determined by Fridewald’s equation), insulin (determined using fluoroimmunoassay technique), glycated HbA1c (determined using high pressure liquid chromatography) and glucose (determined enzymatically by the hexokinase/G6.PDH method).

### Socio-demographic and Behavioral Covariates

Socio-demographic covariates included sex, age (categorized in age groups of 19–29, 30–39, 40–49, 50–59, 60–72 years) and education (categorized in basic (≤1 year), short (1–3 years), long-term (>3 years) self-reported vocational training).

Behavioral covariates included self-reported smoking (binary categorized into current smoker and not current smoker), alcohol consumption (binary categorized by whether Danish weekly drinking limits (no more than 14 drinks for men; no more than 7 drinks for women) were met or exceeded), diet (obtained from a 48-item food-frequency questionnaire [24] and classified into three group of diets: imprudent (i.e., low fruit/vegetables/fish and high fat diet), moderately prudent (i.e., medium fruit/vegetables/fish/fat diet) and prudent (i.e., high fruit/vegetables/fish and low fat diet)). This diet score classification has been validated as a measure of dietary quality in a Danish population [24]. Self-reported weekly moderate to vigorous physical activity, MVPA (h/week) was derived from the PAS2 questionnaire [21], and was included as a continuous variable.

### Study Sample

2544 participants from a total sample of 3471 from Health2006 were included in the analyses. Reasons for exclusion of participants were not working (n = 833, 24%), missing data on sitting time (n = 76, 2%) and missing data on work status (n = 18, 0.5%). Work status included all full-time, part-time and voluntary work. One subject was excluded because of visible limitations to mobility.

### Statistical Analysis

Associations between each domain of sitting time and each cardio-metabolic risk factor were explored in adjusted linear regression models. All models included the covariates sex, age, education, smoking, alcohol consumption, diet and MVPA. Only participants with complete data were included in modeling. Restricted linear splines were performed for all analyses of continuous sitting time variables (*leisure*; *occupational*; *overall*) to account for potential non-linear associations, using knots at the 10, 50 and 90 percentiles, of sitting time. Fit was compared using an F-test. MVPA was included in all models as a restricted linear spline function. When necessary, risk factors were log transformed to yield normal distribution and results were back-transformed.

Participants in diabetic and/or lipid-lowering treatment (n = 164) were excluded from analyses with non-adiposity outcomes (i.e., total cholesterol, HDL cholesterol, LDL cholesterol, triglycerides, insulin, HbA1c, plasma glucose). To consider potential indirect effects by mediation through adiposity, all regression models of non-adiposity outcomes were repeated with adjustment for waist circumference. All results are presented for men and women together, because no evidence of consistent and meaningful differences in associations by sex was found when including interaction terms with sex in all models. P-values below 0.05 were considered statistically significant. Statistical analyses were performed with software package SAS version 9.3.

## Results

The baseline characteristics of the study population by domain of sitting time, socio-demographic and behavioral factors, and cardio-metabolic risk factors are presented in table 1. The mean daily hours of sitting were 3.1 (±1.4) in leisure-time, 4.1 (±2.7) during work, and 7.2 (±2.8) for a total day. Men had higher leisure-time sitting (approximate 18 minutes) than women.

**Table 1:** Baseline characteristics of the study population by sex, domains of sitting time, covariates and cardio-metabolic risk factors (Percentage frequencies (n values) and Means (SD, standard deviations)).

		All		Men		Women	
*N*		2544		1174		1370	
SITTING TIME (hours/day)							
Leisure-time, mean (SD)	–		3.1 (1.4)	–	3.2 (1.4)	–	2.9 (1.3)
Occupational, mean (SD)	–		4.1 (2.7)	–	4.1 (2.8)	–	4.1 (2.5)
Overall, mean (SD)	–		7.2 (2.8)	–	7.3 (3.0)	–	7.1 (2.7)
Categorical, % (n)*	Low Leisure/Low Occupational –		38.4 (977)	–	35.4 (416)	–	40.9 (561)
	High Leisure/Low Occupational		24.1 (612)		26.5 (311)		22.0 (301)
	Low Leisure/High Occupational		26.4 (673)		26.3 (309)		26.5 (364)
	High Leisure/High Occupational		11.1 (282)		11.7 (138)		10.5 (144)
Age (years), % (n)	19–29		9.7 (247)		7.8 (92)		1.3 (155)
	30–39		17.4 (442)		17.2 (202)		17.5 (240)
	40–49		31.6 (805)		30.3 (356)		32.7 (449)
	50–59		29.7 (757)		29.7 (349)		29.8 (408)
	60–72		11.5 (293)		14.9 (175)		8.6 (118)
	Mean (SD)		45.8 (1.3)		46.8 (11.4)		44.9 (11.2)
Education (vocational training years), % (n)	≤1	*n = *2488	16.6 (414)	n = 1149	16.2 (186)	*n = *1339	17.0 (228)
	1–3		34.7 (864)		20.4 (234)		47.0 (630)
	>3		48.6 (1210)		63.4 (729)		36.0 (481)
	Mean (SD)		3.2 (2.1)		3.6 (2.3)		2.9 (1.9)
Smoking, % (n)	Current smoker	*n = *2534	21.5 (546)	*n = *1173	20.5 (240)	*n = *1361	22.5 (306)
	Not current smoker		78.5 (1988)		79.5 (933)		77.5 (1055)
Alchol consumption, (drinks/week), % (n) **	≤ Drinking limits	*n = *2482	72.0 (1788)	*n = *1164	71.0 (827)	*n = *1318	72.9 (961)
	> Drinking limits		27.9 (694)		28.9 (337)		27.1 (357)
	Mean (SD)		8.5 (9.3)		11.6 (11.0)		5.8 (6.3)
Diet, % (n)	Prudent	*n = *2526	24.3 (614)	*n = *1171	16.4 (192)	*n = *1355	31.3 (422)
	Moderately prudent		69.2 (1750)		73.7 (863)		65.4 (887)
	Imprudent		6.4 (162)		9.9 (116)		3.4 (46)
MVPA (hours/week), mean (SD)		–	4.3 (3.9)	–	4.8 (4.3)	–	3.9 (3.6)
Waist circumference (cm), mean (SD)		*n = *2544	87.9 (13.6)	*n = *1174	94.4 (11.9)	*n = *1370	82.4 (12.5)
Body mass index, BMI (kg/m^2^), mean (SD)		*n = *2542	25.8 (4.7)	*n = *1174	26.4 (4.1)	*n = *1368	25.2 (5.0)
Body fat (%), mean (SD)		*n = *2538	29.2 (9.1)	*n = *1172	23.2 (6.6)	*n = *1366	34.3 (7.7)
Total cholesterol (mmol/l), mean (SD)		*n = *2528	5.3 (1.0)	*n = *1169	5.3 (1.0)	*n = *1359	5.2 (1.0)
HDL cholesterol (mmol/l), mean (SD)		*n = *2528	1.5 (0.4)	*n = *1169	1.3 (0.3)	*n = *1359	1.7 (0.4)
LDL cholesterol (mmol/l), mean (SD)		*n = *2509	3.2 (0.9)	*n = *1153	3.3 (0.9)	*n = *1356	3.1 (0.9)
Triglycerides (mmol/l), mean (SD)		*n = *2528	1.3 (1.1)	*n = *1169	1.5 (1.4)	*n = *1359	1.1 (0.6)
Insulin (pmol/l), mean (SD)		*n = *2527	43.7 (34.2)	*n = *1169	46.6 (37.8)	*n = *1358	41.2 (30.5)
Hemoglobin A1c, HbA1c (%), mean (SD)		*n = *2535	5.4 (0.4)	*n = *1169	5.4 (0.4)	*n = *1366	5.4 (0.4)
Plasma glucose (mmol/l), mean (SD)		*n = *2528	5.4 (0.8)	*n = *1169	5.6 (0.8)	*n = *1359	5.3 (0.7)

Table footnotes:

MVPA: Moderate to vigorous physical activity.

* Cut points at ≤3 and >3 h/day for leisure-time sitting, and <6 and 6≥ h/day for occupational sitting, was used.

** Alcohol consumption defined by whether Danish weekly drinking limits (no more than 14 drinks for men; no more than 7 drinks for women) were met or exceeded.

**Figure 1:**
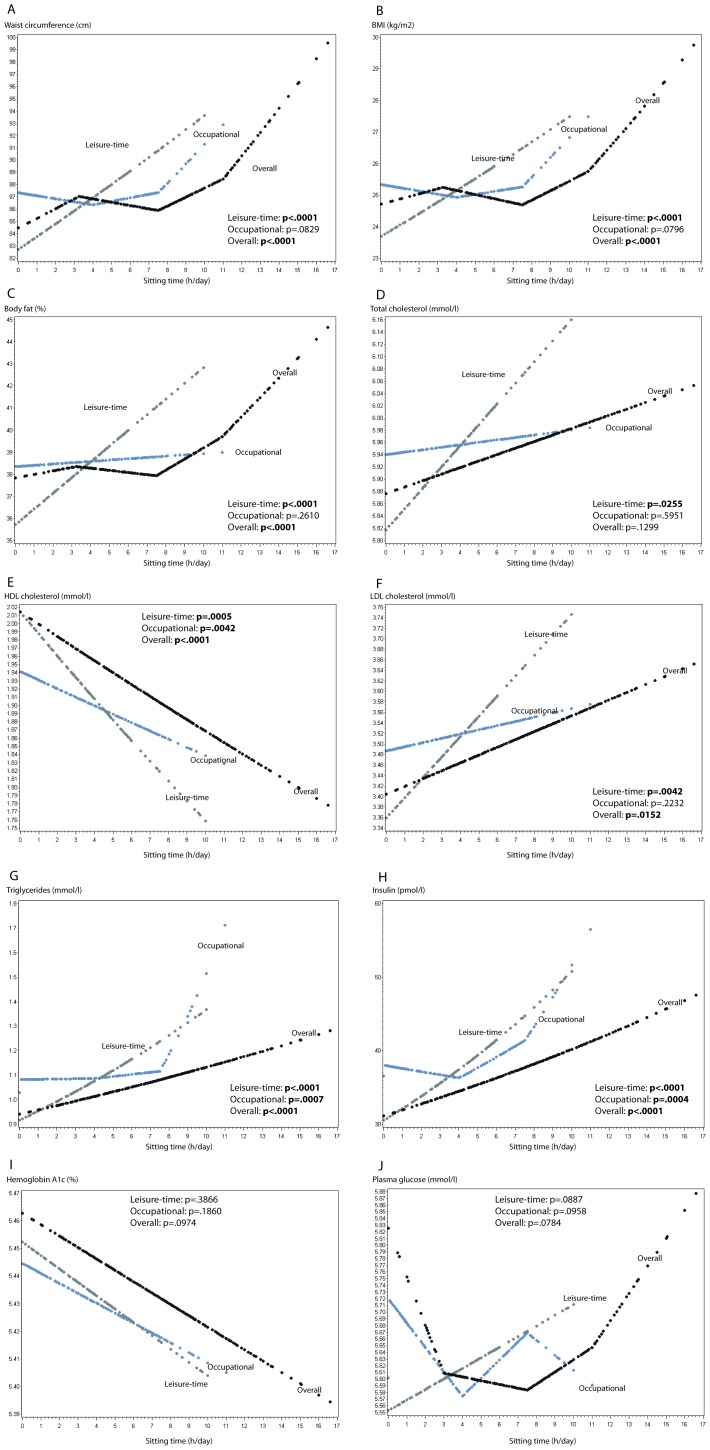
Regression plots for cardio-metabolic risk factors for leisure-time sitting, occupational sitting and overall sitting time. A Waist circumference (n=2414), B BMI (n=2412), C Body fat (n=2408), D Total cholesterol (n=2402), E HDL cholesterol (n=2269), F LDL cholesterol (n=2251), G Triglycerides (n=2269), H Insulin (n=2268), I Hemoglobin A1c (n=2273), J Plasma glucose (n=2269). P-values reported are for the effect of each sitting time domain. All p-values are rounded to four decimals. For non-linear associations restricted linear spline were used with knots at the 10, 50 and 90 percentiles of sitting time for each domain. All models included the covariates sex, age, education, smoking, alcohol consumption, diet and moderate to vigorous physical activity (MVPA). Color legend for all plots: Grey=Leisure-time sitting, Blue=Occupational sitting time, Black=Overall sitting time.

Results of the regression analyses for the continuous sitting time variables (*leisure*; *occupational*; *overall*) are visually represented for all risk factors (Figure 1 A–J): *Leisure-time sitting* had a significant (p<.05) detrimental linear association with all cardio-metabolic risk factors, with the two exceptions of HbA1c (Fig. 1I) and plasma glucose (Fig. 1J). Likewise, *occupational sitting time* was significantly detrimentally associated with HDL cholesterol (direction is inverse with lower levels of “good” HDL cholesterol) (Fig. 1E), triglycerides (Fig. 1G) and insulin (Fig. 1H). By large, peak points in curves were observed at the chosen knots of ±4 h and ±7.5 h (See fig. 1A, B, G, H, J for characteristic of shape) for those associations represented by spline function. *Overall sitting time* was statistical significant for all risk factors, except total cholesterol (Fig. 1D), HbA1c (Fig. 1I) and plasma glucose (Fig. 1J). Curve peak points were observed at the knots placed at ±3.5 h, ±7.5 h and ±11.5 h (Fig. 1A, B, C, J) for those associations represented by spline function.

Model parameter estimates for the *categorical* high/low measure of overall sitting time are reported in table 2. Compared to the reference category Low Leisure-time/Low Occupational sitting, Low Leisure-time/High Occupational was for all risk factors insignificant, with the single exception of insulin. On the whole, estimated parameters followed a same trend across categories: From smallest to greatest estimates are 1) Low Leisure-time/Low Occupational (reference), 2) Low Leisure-time/High Occupational, 3) High Leisure-time/Low Occupational, 4) High Leisure-time/High Occupational. Exceptions are total cholesterol and LDL cholesterol, where risk of Low Leisure-time/High Occupational sitting time was less than the reference, and HbA1c and plasma glucose, where no significant associations were found across all four categories.

**Table 2:** Estimated parameters for the categorical high/low measure of overall sitting time (Beta-estimates (ß), p-values, 95% confidence intervals (CI), and Type III p-values).

CARDIO-METABOLIC RISK FACTORS	CATEGORICAL OVERALL SITTING TIME				
	Low Leisure/Low Occupational	High Leisure/Low Occupational	Low Leisure/High Occupational	High Leisure/High Occupational	Type III p-value
	*Reference*				
Waist circumference (cm), mean (SD)	*0*	*ß:1.0275*	*ß: 1.0065*	*ß: 1.0520*	***p<.0001***
		***p<.0001***	*p:.3113*	***p<.0001***	
		*CI: 1.0139–1.0413*	*CI: 0.9939–1.0194*	*CI: 1.0342–1.0701*	
Body mass index, BMI (kg/m^2^), mean (SD)	*0*	*ß: 1.0296*	*ß: 1.0021*	*ß: 1.0629*	***p<.0001***
		***p:.0010***	*p:.8001*	***p<.0001***	
		*CI: 1.0119–1.0476*	*CI: 0.9857–1.0187*	*CI: 1.0395–1.0868*	
Body fat (%), mean (SD)	*0*	*ß: 1.4853*	*ß: 0.1784*	*ß: 2.8558*	***p<.0001***
		***p<.0001***	*p:.6049*	***p<.0001***	
		*CI: 0.7709–2.1997*	*CI: −0.4977–0.8545*	*CI: 1.9388–3.7728*	
Total cholesterol (mmol/l), mean (SD)	*0*	*ß: 0.0333*	*ß:−0.0207*	*ß: 0.1410*	*p:.1269*
		*p:.5268*	*p:.6774*	***p:.0361***	
		*CI: −0.0698–0.1365*	*CI: −0.1182–0.0768*	*CI: 0.009–0.2728*	
HDL cholesterol (mmol/l), mean (SD)	*0*	*ß: 0.9612*	*ß: 0.9776*	*ß: 0.9266*	***p<.0001***
		***p:.0028***	*p:.0675*	***p<.0001***	
		*CI:0.9366–0.9864*	*CI:0.9541–1.0016*	*CI: 0.8961–0.9581*	
LDL cholesterol (mmol/l), mean (SD)	*0*	*ß: 0.0466*	*ß: −0.0140*	*ß: 0.1893*	***p:.0074***
		*p:.3159*	*p:.7462*	***p:.0015***	
		*CI: −0.0443–0.1371*	*CI: −0.0985–0.0706*	*CI: 0.0723–0.3062*	
Triglycerides (mmol/l), mean (SD)	*0*	*ß: 1.0904*	*ß: 1.0486*	*ß: 1.1989*	***p<.0001***
		***p:.0011***	*p:.0562*	***p<.0001***	
		*CI: 1.0352–1.1486*	*CI: 0.9987–1.1009*	*CI: 1.1211–1.2821*	
Insulin (pmol/l), mean (SD)	*0*	*ß: 1.1267*	*ß: 1.1027*	*ß: 1.2930*	***p<.0001***
		***p:.0004***	***p:.0020***	***p<.0001***	
		*CI: 1.0544–1.2038*	*CI:1.0364–1.1733*	*CI:1.1871–1.4084*	
Hemoglobin A1c, HbA1c (%), mean (SD)	*0*	*ß: 0.9997*	*ß: 0.9991*	*ß: 0.9953*	*p:.7397*
		*p:.9209*	*p:.7812*	*p:.2849*	
		*CI: 0.9931–1.0062*	*CI:0.9929–1.0053*	*CI:0.9869–1.0039*	
Plasma glucose (mmol/l), mean (SD)	*0*	*ß: 0.9971*	*ß: 1.0020*	*ß: 1.0086*	*p:.5256*
		*p:.6144*	*p:.7024*	*p:.2446*	
		*CI: 0.9859–1.0084*	*CI: 0.9916–1.0126*	*CI: 0.9941–1.0234*	

Table footnotes:

All estimates are rounded to four decimals.

Models included the covariates sex, age, education, smoking, alcohol consumption, diet and moderate to vigorous physical activity (MVPA).

In models with adjustment for waist circumference, associations were diluted, but were not essentially changed. Only for insulin were the associations diluted to the null (results not presented). Further, adjustment for leisure-time sitting was performed in analyses with occupational sitting, and for occupational sitting in analyses with leisure-time sitting to account for the possible interrelation between the two sitting time domains. In analysis with occupational sitting, associations weakened slightly after adjustment for leisure-time sitting, otherwise little changed (results not presented). In sensitivity analyses, we excluded participants with >8 h leisure-time sitting <0.5 h, and occupational sitting time >9 h respectively, and results were unchanged. We supplementary adjusted all models for the potential influence of occupational physical activity to account for the possible confounding effect of heavy labor; still results were unchanged. Furthermore, seasonal variation in hours spend sitting across domains was investigated but did neither change the direction nor the magnitude of the associations.

## Discussion

In this population-based study among working adults we found an overall detrimental association between sitting time in any domain and cardio-metabolic risk factors after adjustment for sex, age, education, smoking, alcohol consumption, diet and MVPA. Though, the separate associations were fewer and weaker for occupational sitting time compared to leisure-time sitting. Yet we found a larger detrimental association for categories of high sitting time during both work and leisure-time. To our knowledge, this is the first study to contrast the deleterious associations of prolonged occupational and leisure-time sitting, both separately and jointly.

The identified discrepancy between leisure-time and occupational sitting was rather surprising given the emerging focus on reducing occupational sitting. However, results are consistent with studies conducted in women [8], [25], and with a study of associations with obesity [15]. Findings also match those of Pereira et al. 2012 [13], who correspondingly found less pronounced associations for work sitting than for television viewing with risk factors for cardiovascular disease and diabetes in mid-adulthood. Finally, our results match those of Stamatakis et al. 2012 [14], who finds more consistent associations for television viewing time than for other recreational sitting and occupational sitting or standing (while not confirmed when using objective measures); however, it must be noticed that occupational sedentary time was defined as time spent sitting – and standing – while at work.

Several possible explanations for the identified discrepancy between leisure-time and occupational sitting might be plausible: It may be that work sitting comprises more accompanying breaks than leisure-time sitting. This would be in line with ‘the breaks theory’ suggesting that breaks, even short in duration and light in intensity, have beneficial health effects, counting metabolic associations as discovered by Healy et al. 2011 [26]. As such, different sedentary behaviors may have different correlates, just like MVPA, and the stronger associations for leisure-time sitting may reflect actual lower energy expenditure for this behavior. Furthermore, what we do while sitting, for instance snacking [27], could be an associated aspect. Mechanisms underlying our findings are thus likely to be complex.

When looking at specific risk factors, HbA1c and plasma glucose were consistently not found related with any sitting time domain (*leisure*, *occupational*, *overall*, *categorical*). Pereira et al. 2012 [13] also looked at HbA1c, and found no associations with work sitting and television viewing, alike our results. We found that insulin, marker of glucose homeostasis, was associated much better with all sitting domains. Insulin together with triglycerides and HDL cholesterol was associated with sitting time regardless of the continuous sitting time domains, pointing to possible important determinants and warranting further investigation. However, the association between sitting time and insulin was strongly diluted when waist circumference was adjusted for.

The fact that waist circumference diluted the associations suggests that adiposity may function as a pathway through which sitting time affect health for some risk factors – as also suggested by others [28]–[30]. In line with Chau and colleagues (2012) [15] who showed a clear positive association between leisure-time sitting and obesity risk while the association with occupational sitting was less distinct, our results also indicate a less clear association between occupational sitting and adiposity-related outcomes; insignificant for both BMI, waist circumference and body fat.

In contrast to other studies 13,31 we did not find any consistent sex differences. Effect modification between sex and each domain of sitting time on cardio-metabolic risk factors was explored by including interaction terms in all models. Meanwhile, there was no evidence of consistent and meaningful differences in associations by sex, and results were thus presented for men and women together.

A main strength of the present study is that we used a detailed physical activity questionnaire specifying different types of sedentary activities performed and the duration of each. That is, leisure-time was not limited to television viewing and the sitting domains were of continuous form, thus more predictive information was retained than when considered as categorical variables only, which frequently is the case for self-reported sedentary behavior instruments.

Also, participants included part-time workers and workers doing voluntary work, and thus we were not restricted to subjects in paid employment, in contrast to Pereira et al. 2012 [13], or to full-time workers as in Chau et al. 2012 [15]. Furthermore, we adjusted for dietary habits, the lack of which has been identified as a main drawback of many studies within the field [3]. Another main study strength is the sensitivity analyses performed. Based on these, we believe our findings appear rather robust.

Yet, some limitations of this study need to be addressed, one of which is that the information on sitting time was based on self-assessment, which entail some degree of misclassification. It is possible that some participants have answered the question on occupational sitting time with their daily working hours. A further limitation could be that the leisure-time sitting question does not explicitly refer to leisure-time Internet and computer use, a sitting behavior of rapid proliferation. Also, socializing (chat, phone, texts) may be a significant leisure-time sitting domain. Nonetheless, in validation studies, the PAS2 questionnaire was found to correlate well with physical activity diaries [22] and maximum oxygen uptake [23]. Moreover, the average seven hours of daily sitting reported by the study participants are similar to what others have measured objectively [26], [30]. Besides, while objective measurement devices such as inclinometers and accelerometers can avoid the vagaries of self-report methods, self-report assessment methods are required for information on sitting time domains. Another issue that can be debated is our choice of cut-points for defining the categories of high/low sitting time, which we based on the distribution of the study population and previous used Danish categories [32]. Given the current diversity in cut-points, standardizing may be an important issue for future research, yet sensitive of the specific measurement instruments. Participants with visible limitations to mobility were excluded in the study, and participants in diabetic and/or lipid-lowering treatment were excluded from analyses with non-adiposity outcomes, yet some residual effect of poor health status affecting the hours spend sitting cannot be ruled out. That said our study sample comprises working adults, typically in good health compared with the general population. Finally the cross-sectional nature of this study precludes causal linkage between our sitting time domains and cardio-metabolic risk factors. Thus, a prime question that remains unanswered is the causation between the anthropometric measures and sitting time; do obese people sit more or do people who sit a lot become obese – or does bidirectional causality exist?

Regarding the analyses, a main drawback is the multiple tests conducted, affecting the power. A further drawback is that spline analysis has an innate limitation of over fitting data. Because the peaks and valleys (given the knots applied) along the spline functions were similar across risk factors, it may be interesting to further explore aspects of a potential dose response curve for occupational and overall sitting, such as whether our thresholds are captured correctly. This could have important implications, especially in the scope to make recommendations. It could be that e.g., the identified descending shape up till ±4 h. of occupational sitting is indication of a suitable balance between occupational sitting time and occupational physical activity, drawing parallels to the findings of Holtermann et al. [33, 34] that refute the common belief of all physical activity being good for health, by indicating opposing effects of occupational and leisure-time physical activity on global health [33] and on cardiovascular and all-cause mortality [34]. The same hypothesis can be applied to overall sitting time, with a possible healthy balance of sitting ±3.5 h. to ±7.5 h. a day. That said residual socio-economic impact could also be a plausible explanation.

The purpose of this study was to explore the separate and joint associations of occupational and leisure-time sitting with cardio-metabolic risk factors in a sample of working adults. Our findings add to the evidence that prolonged sitting time is associated with main contributing factors of cardio-metabolic disorders. Yet it also adjoins that occupational sitting time may have a less detrimental association with cardio-metabolic risk factors than leisure-time sitting. Taken today’s literature together, it is unclear how the choice of sitting time domain, or sedentary behavior, may influence health risk. This explorative study contributes to this discussion, and amplifies the need for further focus in this area prior to making assumptions about equivalent health risks across sedentary behaviors. As a final point, developing strategies for reducing sitting during leisure-time may to date have stronger justification than targeting occupational sitting based on the present, albeit cross-sectional, evidence.
